# A Challenging Case of Kaposi Sarcoma Inflammatory Cytokine Syndrome

**DOI:** 10.7759/cureus.42218

**Published:** 2023-07-20

**Authors:** Ammar Al-Obaidi, Himil Mahadevia, Zain Syed, Shahzad Raza

**Affiliations:** 1 Hematology/Oncology, University of Missouri Kansas City, Kansas City, USA; 2 Internal Medicine, University of Missouri Kansas City, Kansas City, USA; 3 Biomedical Engineering, Case Western Reserve University, Cleveland, USA; 4 Hematology and Medical Oncology, The Cleveland Clinic, Cleveland, USA

**Keywords:** rituximab, hhv8, hiv, kaposi sarcoma, kics

## Abstract

Kaposi Sarcoma Inflammatory Cytokine Syndrome (KICS) is a serious, uncommon disease that occurs in patients who are positive for HIV and human herpesvirus-8 (HHV-8). It is characterized by a constellation of clinical findings, including fever, weight loss, and fluid retention, as well as a lack of multicentric Castleman disease (MCD) features on histopathology and an elevated serum HHV-8 viral load. Diagnosis is often delayed, and treatment options are limited, culminating in high mortality rates. We hereby present a 32-year-old male patient with HIV who was untreated for a few years and came with fever, night sweats, pancytopenia, and widespread adenopathy. A thorough evaluation of opportunistic infections was unremarkable. Clinically MCD was suspected, but lymph node biopsy only showed Kaposi sarcoma (KS) with no characteristic features of MCD. However, with clinical deterioration, KICS was strongly suspected. Kaposi sarcoma immune reconstitution syndrome (KS-IRIS) was also a possibility as the patient was restarted on antiretroviral therapy. Rituximab was commenced, but the patient suffered a cardiac arrest and could not be revived. Alternative diagnosis must be explored in patients with HIV presenting with constitutional symptoms, cytopenia, and adenopathy after opportunistic infections and malignancies are ruled out. If they have KS with HHV-8 positivity and there is a lack of characteristic features of MCD in lymph node biopsy, prompt suspicion of KICS should be made, and treatment with rituximab and/or chemotherapy should be instituted rapidly. KS-IRIS is also possible if patients have recently received antiretroviral therapy and have a rapid decline in viral load and increase in CD4 counts (immunological recovery). HHV8 viral load levels may help to distinguish between these two inflammatory conditions.

## Introduction

Kaposi sarcoma inflammatory cytokine syndrome (KICS) is a rare disease that occurs exclusively in patients with a co-infection of human herpesvirus-8 (HHV-8) and HIV [1]. Its clinical presentation is typically similar to multicentric Castleman disease (MCD), which can also occur in HHV-8-positive individuals. KICS can be distinguished from MCD by the absence of characteristic microscopic findings. These include onion skinning of mantle zones, regression of germinal centers, and vascular proliferation with perivascular hyalinization in affected lymph nodes [2]. HHV-8 has latent and lytic phases of infection. Rapid multiplication of virions and expression of hundreds of genes, especially viral homolog of interleukin-6 (IL-6) during the lytic phase, can precipitate the release of more cytokines by the immune system, including human IL-6. This triggers an inflammatory syndrome characterized by clinical findings such as fever, fatigue, edema, arthralgias, gastrointestinal and respiratory symptoms, altered mental status, and neuropathy [1,3,4]. Lab findings include anemia, thrombocytopenia, hypoalbuminemia, and elevated inflammatory markers [1,3,4]. Radiographic findings include adenopathy, hepatosplenomegaly, and body cavity effusions [1,3,4]. Diagnosis is based on a constellation of the above-mentioned clinical findings, evidence of systemic inflammation, elevated HHV-8 viral load, and lack of MCD features on pathology [1,2]. As of now, there are no standard treatment guidelines. A few treatments, such as rituximab plus liposomal doxorubicin and high-dose zidovudine plus valganciclovir, have shown some efficacy [5-7]. Despite these treatments, the morbidity and mortality rates remain quite high, reaching up to 60% [8]. We describe a patient with HIV who had discontinued antiretroviral therapy (ART) for a few years. The patient presented with constitutional symptoms, pancytopenia, hepatosplenomegaly, and widespread lymphadenopathy for over a month. Initial suspicions of opportunistic infections were dismissed when a comprehensive evaluation for infections proved unrevealing. Due to a very low cluster of differentiation 4 (CD4) counts - 90 cells/uL and a high HIV viral load, the patient was started on ART, which included bictegravir (an integrase inhibitor), emtricitabine (a nucleoside reverse transcriptase inhibitor), and tenofovir (a nucleotide reverse transcriptase inhibitor). MCD was suspected, but microscopic analysis of right inguinal lymph node tissue did not reveal characteristic features. It, however, revealed Kaposi sarcoma (KS). There was strong suspicion for KICS, although Kaposi sarcoma immune reconstitution inflammatory syndrome (KS-IRIS) was also a possibility. KS-IRIS occurs in the early phase of ART initiation when there is an increase in the CD4 count leading to recovery of immune function.

## Case presentation

We present the case of a 32-year-old male with a past medical history of HIV who presented with fever and night sweats for over one month. He had exertional shortness of breath and a mild non-productive cough. He had not taken antiretroviral medications for approximately the last three years and had not had any laboratory tests performed for the past two years. He had been prescribed bictegravir, emtricitabine, and tenofovir regimen for HIV. Initial workup revealed significant pancytopenia with leukocytes - 3.28 Th/ul, hemoglobin - 7.1 g/dl, and platelets - 80 Th/ul. CT scan of the abdomen/pelvis revealed marked hepatosplenomegaly and lymphadenopathy predominantly within the iliac chains and periaortic/paracaval lymph nodes. CT angiogram of the chest noted prominent axillary, supraclavicular, and mediastinal lymph nodes and cardiac enlargement with small pericardial effusion. These findings were suggestive of lymphoproliferative disorder versus opportunistic infection. He was started on empiric doxycycline, given his exposure to birds and the possibility of psittacosis. It was initially decided to hold ART until a definitive diagnosis was reached to avoid IRIS. Further, the workup revealed a CD4 count of 90 cells/uL and an HIV-1 RNA quantitative log of 6.37 copies/ml. The urinalysis was unremarkable, and the urine toxicology screen was negative. COVID-19 polymerase chain reaction (PCR) testing also came back negative. Tests for various antibodies, including Antinuclear (ANA), anti-mitochondrial (AMA), anti-smooth muscle actin (SMA), and celiac antibodies, all returned negative results. Similarly, antigen tests for Histoplasma in the urine, strep pneumonia in the urine, and legionella in the urine were negative. We conducted a hepatitis panel and a pneumonia panel, both of which were unrevealing. Respiratory panel PCR testing for various organisms such as chlamydia, mycoplasma, bordetella, metapneumovirus, and parainfluenza virus all returned negative results. We performed a bone marrow biopsy which revealed hypercellularity but no evidence of leukemia or lymphoma. Tests for cytomegalovirus (CMV) and Epstein Barr virus (EBV) using PCR were negative. Blood and urine cultures showed no bacterial growth. Finally, we restarted the patient on his ART. The pneumocystis PCR test came back negative. We performed a right inguinal lymph node biopsy, and both the Acid-fast bacilli (AFB) and Gram stain on the lymph node tissue were negative. Later, we conducted an esophagogastroduodenoscopy (EGD) and colonoscopy to evaluate the patient for probable iron deficiency anemia and to rule out intestinal lymphoma. The results were unremarkable, except for the presence of esophageal candidiasis. For this, we started the patient on fluconazole. The patient had intermittent fevers, so we started him on an anti-Mycobacterium avium complex (MAC) regimen, which included azithromycin and ethambutol. Despite these interventions, the patient continued to have pancytopenia.

**Figure 1:**
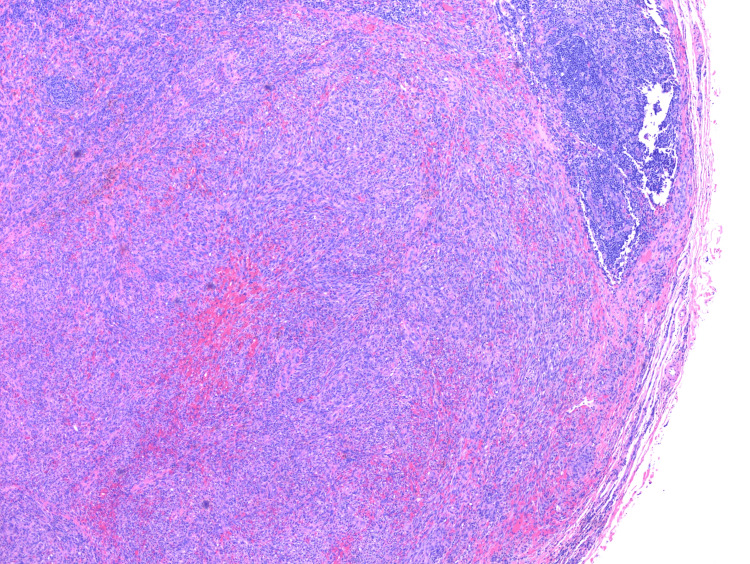
H&E staining of lymph node biopsy sample in 40x resolution. Microscopic image disruption of normal lymph node architecture by Kaposi sarcoma.

**Figure 2:**
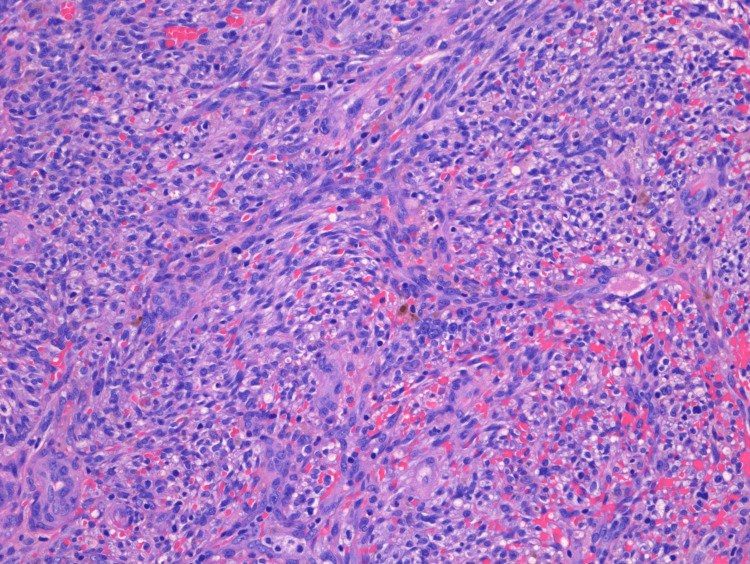
H&E staining of lymph node biopsy sample in 200x resolution. Microscopy image showing Kaposi sarcoma cells with a few regressed germinal centers along with onion skinning.

**Figure 3:**
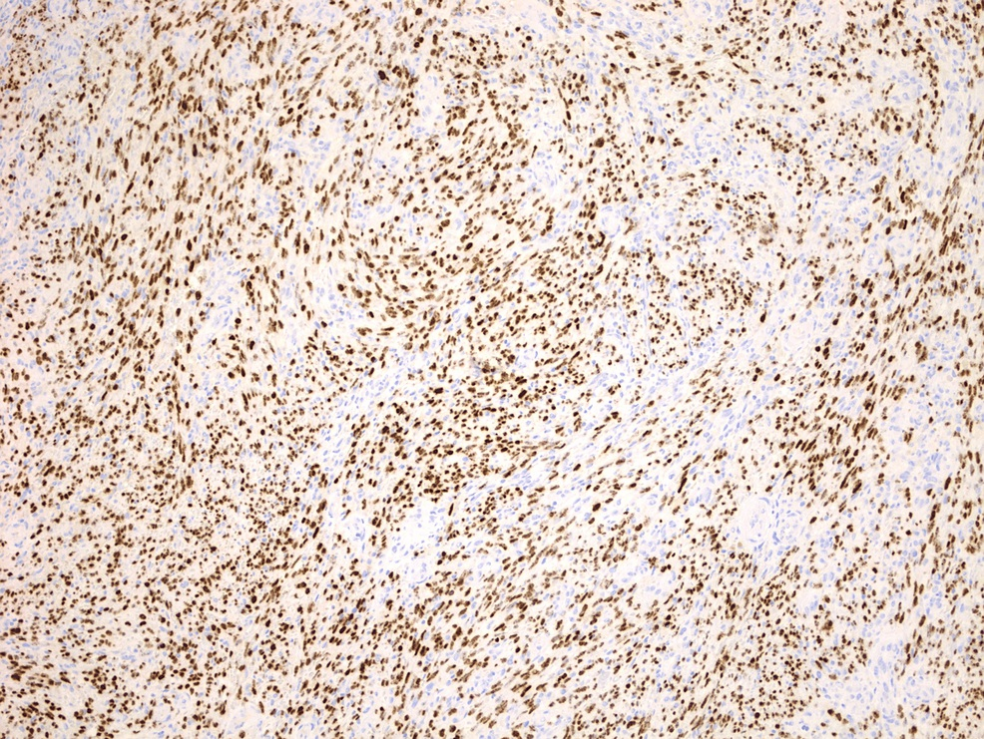
Immunohistochemical staining of lymph node biopsy sample. Microscopy image showing Kaposi sarcoma cells staining positive for human herpesvirus-8.

Clinically, there was also concern for HHV-8-associated MCD. Lymph node biopsy results arrived more than a week later and showed that nodal tissue was largely replaced by KS (Figure 1). The residual nodal tissue showed a couple of regressed germinal centers with onion skinning (Figure 2). However, diagnostic features of MCD were not found in the biopsy sample. Furthermore, HHV-8 antibody testing was performed, and it came back positive (titers>1:20) (Figure 3). He was commenced on valganciclovir. The patient was advised to follow up outpatient for rituximab therapy for KS and probable MCD. In the interim, the patient continued to experience fevers, which were attributed to KS. However, since the patient had been on ART for a couple of weeks, there was also concern for IRIS. Liver function tests showed elevated bilirubin (2.2 mg/dl) and alkaline phosphatase (ALP) (217 U/L). We considered that this could be due to cholestasis, medication effects, or possible MCD. Ultrasound of the abdomen showed thickened gallbladder with no stones. In a few days, bilirubin levels worsened (4 mg/dl), and the patient developed acute kidney injury (creatinine - 1.4 from baseline 0.8). Due to concern for KS-IRIS, the National Institutes of Health (NIH) treatment guidelines were reviewed, which recommended adding chemotherapy to the ART regimen instead of steroids. However, given the patient's clinical condition, we decided to initiate steroid treatment, continue the ART regimen, and start rituximab earlier than the standard six weeks. This was due to concerns about the patient's ability to tolerate chemotherapy.

There was also a potential concern about KICS accounting for the patient's presentation. With further worsening of liver and kidney function and persistent pancytopenia, it was finally decided to start rituximab and dexamethasone immediately and commence liposomal doxorubicin (lipo-dox) in addition as well. The patient was explained the risk of toxicity (including death) in the setting of organ dysfunction, and the patient consented. The patient was administered 40 mg of IV dexamethasones. Rituximab infusion was commenced concomitantly, and if he tolerated it well, then lipo-dox was to be administered. During rituximab infusion, the patient developed a severe hypersensitivity reaction. Hypersensitivity protocol was initiated, and once the vitals were stabilized, rituximab was restarted at half the rate. Soon after, the patient went into cardiac arrest with pulseless electrical activity (PEA). Resuscitation measures were initiated and performed for almost an hour, but the patient could not be brought back. Labs revealed severe hyperkalemia. However, anaphylaxis-induced cardiac arrest was also a possibility.

## Discussion

KICS is a newly recognized rare disease entity that occurs in patients co-infected with HIV and HHV-8 and is characterized by systemic inflammation and signs and symptoms resembling MCD without the characteristic pathology on lymph node biopsy [8]. One of the most striking features is elevated serum HHV-8 levels (>1000 copies/ml) [4]. The diagnostic criteria are the presence of at least two manifestations from at least two categories (including clinical symptoms, laboratory tests, and imaging), evidence of systemic inflammation, elevated viral load, and lack of features of MCD on biopsy [1,4]. Biopsy in KICS may show KS infiltration or reactive proliferation of cells [8]. It usually occurs in HIV-positive patients who are not treated appropriately and have very high HIV viral loads and very low CD4 counts (<100 cells/uL) [5]. IL-6 and IL-10 levels are often elevated as well in patients with KICS. In a retrospective study of patients with KICS, MCD, and KS, cytokine levels of viral IL-6, human IL-6, IL-10, and HHV-8 viral load were substantially higher in KICS as well as MCD patients compared to patients with KS only [3]. These findings were corroborated in a prospective study as well [1]. Thus, KICS is probably due to the abnormally high proliferation of HHV-8 in plasmablasts (lytic phase) and abnormally high production of viral IL-6, which drives a pro-inflammatory state by stimulating human IL-6 and other cytokines [4]. Bone marrow biopsy was negative for lymphoma or leukemia in our patient. Bone marrow biopsy in KICS may show increased cellularity, reactive plasmacytosis, and HHV-8-infected plasma cells [8]. However, the bone marrow biopsy did not reveal such findings in our patient. A lymph node biopsy was performed, which did not show any characteristic features of MCD [2]. KS-IRIS is quite similar to KICS in clinical presentation and laboratory findings [9,10]. It is characterized by the progression of existing lesions of KS, the development of new lesions, and new organ system involvement [8]. Lymph node biopsy may show typical KS findings like vascular channels, spindled cells, macrophages with hemosiderin, and extravasated erythrocytes [8]. Importantly, HHV-8 viral load levels are low, which is a key differentiating finding from KICS [10]. It has a temporal association with ART administration and usually occurs when CD4 counts are too low and rise by more than 50 cells/uL with therapy [9]. Predictors of KS-IRIS include clinical KS before ART, less than 30% hematocrit, high pre-ART HIV viral load, and detectable pre-ART serum HHV-8 DNA [11]. Our patient had a hematocrit of 23% and a very high HIV viral load of 2,370,000 copies/ml. Additionally, the patient showed probable new organ system involvement, as suggested by abnormal liver function tests conducted after initiating ART. However, the patient did not have any clinically evident KS lesions before starting ART. We did not measure the HHV-8 viral DNA load in our patient or re-measure the CD4 count and HIV viral load after a few weeks of ART. As a result, it remained unclear whether the ART had suppressed the viral load and led to immune reconstitution. Most importantly, the patient's clinical symptoms and laboratory markers continued to worsen both before and after initiating ART. There was no acute worsening of anemia and thrombocytopenia a few weeks after ART. Fevers were persistent in the patient, and there was no acute exacerbation after the onset of ART. It is challenging to differentiate KICS from KS-IRIS, especially when we cannot re-measure HIV viral load, CD4 count, and HHV-8 viral DNA. Although, we believe the case presentation was more consistent with KICS, with KS-IRIS also being a somewhat less likely possibility.

Tally T et al. described an HIV-positive patient initially suspected of sepsis [12]. Further, the workup revealed HHV-8 positive status and KS [12]. The patient was ultimately diagnosed with KICS, and despite getting the appropriate treatment, the patient passed away [12]. This highlights the fact that KICS may easily be confused with sepsis. The only definite way to differentiate MCD from KICS is the lack of MCD characteristic features in lymph node tissue samples [3]. However, studies have shown that KICS often does not have extensive lymphadenopathy and splenomegaly, which is seen in MCD. Furthermore, KICS more often occurs in HIV-positive patients who have dramatically low CD4 counts (less than 100 copies/uL) and uncontrolled HIV viremia. In contrast, MCD more frequently occurs when CD4 counts are not less than 200 copies/uL and HIV viral load is somewhat suppressed [13]. Mortality rates are very high for KICS. A prospective study demonstrated 60% mortality in patients with KICS [5]. Treatment options for KICS are very limited. MCD and KICS have concurring pathophysiology, and thus the management for KICS is approached similarly to MCD. In a phase II study of 24 MCD patients by Gérard L et al., 92% of patients had sustained resolution of symptoms in 60 days with rituximab [14]. However, KS worsened in more than one-third of the participants [14]. This was because rituximab-induced depletion of B lymphocytes compromised immune incursion against KS. Adding liposomal doxorubicin, a front-line chemotherapeutic agent for KS, to rituximab led to excellent responses in more than 90% of the patients in another study [6]. Only one out of 17 patients had accentuation of KS with this combination approach [6]. A total of 14 HIV-positive patients with HHV-8 MCD were treated with a seven-day regimen of high-dose zidovudine and valganciclovir [7]. Three patients showed complete responses, and one had a partial response. The disease remained stable in nine patients, while one patient had progressive disease [7]. Most patients experienced pancytopenia and gastrointestinal side effects [7]. IL-6 antagonists such as siltuximab and tocilizumab, which are used in MCD and have shown some clinical responses [15], also appear to play a role in KICS. However, these IL-6 antagonists have not yet been evaluated for their efficacy in treating KICS.

## Conclusions

KICS is a rare under-recognized severe disease with high mortality. It usually occurs in HIV-infected patients who are co-infected with HHV-8. Symptoms and clinical presentation often mimic opportunistic infection/sepsis. In patients who are rapidly deteriorating clinically, have no infectious cause identified, and show a lack of response to antibiotics, KICS should be considered one of the differentials. The only way to differentiate it from clinically similar MCD is lymph node biopsy showing a lack of characteristic MCD features. KICS and KS-IRIS have very overlapping findings. KS-IRIS is associated with the initiation of ART and occurs when CD4 counts have improved, and HIV loads have been suppressed. One way to distinguish is by measuring HHV-8 viral load. It is very high in patients with KICS but low in patients with KS-IRIS. Treatment with rituximab and lipo-dox may help in the treatment of KICS. However, despite treatment, mortality rates are high. More insight into the pathophysiology of KICS may help us to form better treatments. More prospective studies evaluating currently used drugs like rituximab and new drugs like IL-6 antagonists should be conducted for KICS.
